# Respiratory and haemodynamic changes during decremental open lung positive end-expiratory pressure titration in patients with acute respiratory distress syndrome

**DOI:** 10.1186/cc7786

**Published:** 2009-04-17

**Authors:** Christian Gernoth, Gerhard Wagner, Paolo Pelosi, Thomas Luecke

**Affiliations:** 1 Department of Anesthesiology and Critical Care Medicine, University Hospital Mannheim, Faculty of Medicine, University of Heidelberg, Theodor-Kutzer Ufer, 68165 Mannheim, Germany; 2 Department of Anesthesiology an Critical Care Medicine, Robert-Bosch Hospital, Auerbachstrasse 110, 70376 Stuttgart, Germany; 3 Department of Ambient, Health and Safety, University of Insubria, c/o Villa Toeplitz Via G.B. Vico, 46 21100 Varese, Italy

## Abstract

**Introduction:**

To investigate haemodynamic and respiratory changes during lung recruitment and decremental positive end-expiratory pressure (PEEP) titration for open lung ventilation in patients with acute respiratory distress syndrome (ARDS) a prospective, clinical trial was performed involving 12 adult patients with ARDS treated in the surgical intensive care unit in a university hospital.

**Methods:**

A software programme (Open Lung Tool™) incorporated into a standard ventilator controlled the recruitment (pressure-controlled ventilation with fixed PEEP at 20 cmH_2_O and increased driving pressures at 20, 25 and 30 cmH_2_O for two minutes each) and PEEP titration (PEEP lowered by 2 cmH_2_O every two minutes, with tidal volume set at 6 ml/kg). The open lung PEEP (OL-PEEP) was defined as the PEEP level yielding maximum dynamic respiratory compliance plus 2 cmH_2_O. Gas exchange, respiratory mechanics and central haemodynamics using the Pulse Contour Cardiac Output Monitor (PiCCO™), as well as transoesophageal echocardiography were measured at the following steps: at baseline (T_0_); during the final recruitment step with PEEP at 20 cmH_2_O and driving pressure at 30 cmH_2_O, (T_20/30_); at OL-PEEP, following another recruitment manoeuvre (T_OLP_).

**Results:**

The ratio of partial pressure of arterial oxygen (PaO_2_) to fraction of inspired oxygen (FiO_2_) increased from T_0 _to T_OLP _(120 ± 59 versus 146 ± 64 mmHg, *P *< 0.005), as did dynamic respiratory compliance (23 ± 5 versus 27 ± 6 ml/cmH_2_O, *P *< 0.005). At constant PEEP (14 ± 3 cmH_2_O) and tidal volumes, peak inspiratory pressure decreased (32 ± 3 versus 29 ± 3 cmH_2_O, *P *< 0.005), although partial pressure of arterial carbon dioxide (PaCO_2_) was unchanged (58 ± 22 versus 53 ± 18 mmHg). No significant decrease in mean arterial pressure, stroke volume or cardiac output occurred during the recruitment (T_20/30_). However, left ventricular end-diastolic area decreased at T_20/30 _due to a decrease in the left ventricular end-diastolic septal-lateral diameter, while right ventricular end-diastolic area increased. Right ventricular function, estimated by the right ventricular Tei-index, deteriorated during the recruitment manoeuvre, but improved at T_OLP_.

**Conclusions:**

A standardised open lung strategy increased oxygenation and improved respiratory system compliance. No major haemodynamic compromise was observed, although the increase in right ventricular Tei-index and right ventricular end-diastolic area and the decrease in left ventricular end-diastolic septal-lateral diameter during the recruitment suggested an increased right ventricular stress and strain. Right ventricular function was significantly improved at T_OLP _compared with T_0_, although left ventricular function was unchanged, indicating effective lung volume optimisation.

## Introduction

Cyclical opening and closing of atelectatic alveoli and distal small airways with tidal ventilation is known to be a basic mechanism leading to ventilator-induced lung injury (VILI) [1]. To prevent alveolar cycling and derecruitment in acute lung injury (ALI) and acute respiratory distress syndrome (ARDS), high levels of positive end-expiratory pressure (PEEP) have been proposed to counterbalance the increased lung mass resulting from oedema, inflammation and infiltration, and to maintain normal functional residual capacity [2]. Although higher levels of PEEP have been shown to prevent VILI in animal studies [1,3], the random application of either higher or lower levels of PEEP in an unselected population of patients with ALI/ARDS did not significantly improve outcome in three large randomised trials [4-6]. It has been argued that in a partially collapsed lung, high levels of PEEP alone could result in only limited lung protection [4] while exerting its negative effects [7,8]. Therefore, the 'open lung concept' has been proposed [9], aimed at opening up all recruitable alveoli by applying high inflation pressures (lung recruitment manoeuvre (RM) to 'open up the lung'). Once the lung is thought to be recruited, the open lung PEEP (OL-PEEP) is defined as the level of PEEP that prevents end-expiratory collapse ('to keep the lung open'). A decremental PEEP trial after full lung recruitment allows for PEEP titration along the deflation limb of the pressure/volume curve while observing changes in both oxygenation and respiratory mechanics [10,11]. During a decremental PEEP trial, the point of maximum curvature and maximal tidal respiratory compliance have been shown to correspond to OL-PEEP in theoretical and animal models of ALI/ARDS [10,12,13].

However, high intrathoracic pressures applied during lung recruitment and PEEP titration may cause barotrauma or haemodynamic instability [8,14-16], representing a potential limitation of the open lung concept. In particular lung recruitment is known to result in significant haemodynamic compromise because of an acute right ventricular pressure overload, with an acute leftward septal shift in transoesophageal echocardiography [14,16,17]. On the other hand, re-establishing 'normal' functional residual capacity (FRC) by optimum PEEP should result in unloading of the right ventricle, as pulmonary vascular resistance is related to lung volume in a bimodal fashion, with resistance to flow being minimal near FRC [18]. In addition, recruitment of collapsed alveoli, by increasing regional alveolar partial pressure of arterial oxygen (PaO_2_), should reduce hypoxic pulmonary vasoconstriction and thus pulmonary vasomotor tone [19,20], thereby unloading the right ventricle. Although the potential negative effects of RMs are well defined, it is still unclear whether RMs are beneficial to improve respiratory function when patients with ALI/ARDS are ventilated with high PEEP and low tidal volume, that is using lung protective ventilation.

Therefore, the aims of the present study were to investigate the effects of a standardised, computer-controlled open lung strategy on the respiratory function and haemodynamics in patients with ARDS already being ventilated in a lung protective mode.

## Materials and methods

### Patients

Following approval from the local ethics committee, written informed consent was obtained from the patients' next of kins. Every mechanically ventilated patient with ARDS (lung injury score ≥ 2.5) was considered eligible for the study [21]. Further exclusion criteria were the following: age younger than 18 years, mechanical ventilation for more than 96 hours, pregnancy, severe head injury, aortic or femoral aneurysms, inherited cardiac malformations, presence of arrhythmias, immunosuppression, end-stage chronic organ failure and expected survival less than 24 hours.

Before interventions were started patients had to be haemodynamically stable (described below). Adequate sedation (Richmond agitation sedation scale score -5) [22] was ensured with intravenous midazolam (5 to 15 mg/hour) and fentanyl (0.5 to 2.5 mg/hour) throughout the study. Paralysing agents were not used. The ventilator was set by the attending physician in the pressure-control mode with tidal volumes ranging between 5 to 8 ml/kg ideal body weight (IBW), an inspiration:expiration ratio of 1:1 and respiratory rate (RR) set to keep arterial pH greater than 7.20. PEEP was set during an incremental PEEP trial using the oxygenation response as the primary endpoint.

Improvement in oxygenation was arbitrarily defined as an increase in PaO_2 _exceeding 10 mmHg as described previously [23]. Noradrenaline was used if mean arterial pressure (MAP) was below 65 mmHg despite adequate intravascular volume status. Dobutamine was added in case the cardiac index (CI) was less than 2.5 l/min/m^2^. All patients had a triple-lumen central venous catheter (via the subclavian or internal jugular vein) and a thermodilution catheter (5 F Pulsiocath™, Pulsion Medical Systems, Munich, Germany) via a femoral artery inserted. The Pulse Contour Cardiac Output monitor (PiCCOplus™) was used for haemodynamic measurements and intravascular volume optimisation in all patients as standard care.

### Haemodynamics and intravascular volume measurements

The PiCCO apparatus was calibrated with the intermittent transpulmonary thermodilution technique using three times 20 ml iced saline immediately before the first set of measurements. CI was calculated by the PiCCO monitor from the area under the arterial pulse curve of each heartbeat and from an estimation of systemic vascular resistance based on MAP and a manually entered central venous pressure. Haemodynamic stability was defined as a MAP greater than 65 mmHg, HR less than 130 beats/min and a CI greater than 2.5 l/min/m^2^. Intravascular volume status was titrated using the intrathoracic blood volume index aimed at low normal values (750 to 950 ml/m^2^).

### Transoesophageal echocardiography

According to the recommendations of the American Society of Echocardiography a comprehensive transoesophageal echocardiography (Vivid III, GE, Piscataway, NJ, USA) was conducted to exclude structural cardiac abnormalities or severe valvular heart diseases. For the study, left and right ventricular diameters and function were measured in the transgastric short axis mid-papillary view, the bicaval view was used to measure the ventilation-associated caval differences during the recruitment manoeuvre. The right ventricular Tei index [24,25] was used to assess systolic and diastolic right ventricular function. Right ventricular Tei index is equal to the sum of the isovolumic contraction time and the isovolumic relaxation time, divided by ejection time. It is calculated using the closing interval of the tricuspid valve (pulsed-wave doppler spectra, mid-oesophageal right ventricular inflow-outflow-view) and the opening time of the pulmonary valve (pulsed wave Doppler, view of mid-upper-oesophageal short axis of the ascending aorta). Tei index is a particular useful means of assessing global ventricular function because it is simple and reproducible, independent of ventricular geometry and is not significantly affected by HR, blood pressure or changing ventricular loading conditions [24,25]. Right ventricular end-diastolic and end-systolic diameters were obtained in the transgastric short axis mid-papillary view.

### Respiratory mechanics

Lung recruitment and PEEP titration was guided and standardised using a dedicated software (Open Lung Tool™, Maquet Critical Care AB, Solna, Sweden) incorporated into the Servo-i™ ventilator. The Open Lung Tool™ is a real-time monitoring of the changes in respiratory system compliance during the clinical application of a recruitment strategy. It continuously displays end-inspiratory pressure (EIP), PEEP, inspired and expired tidal volumes and dynamic compliance of the respiratory system (Cdyn). Cdyn was automatically calculated as Vtinsp/EIP – PEEP. The graphical display of Cdyn will indicate the response of the patients' respiratory system mechanics to each change in applied airway pressure.

### Lung recruitment and PEEP titration

The open lung procedure was divided into two distinct parts: the lung recruitment phase and the open lung PEEP titration.

**Figure 1:**
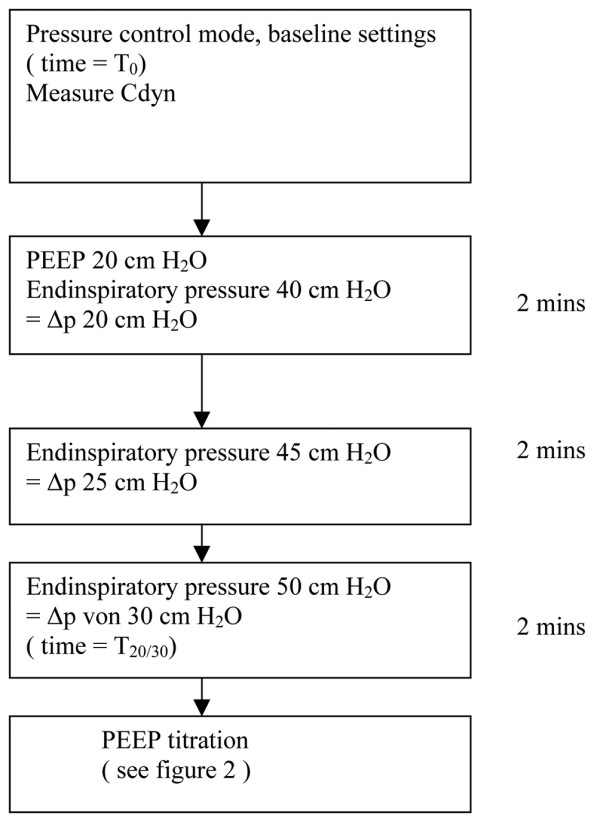
Recruitment procedure using the Open Lung Tool™. Cdyn = dynamic compliance of the respiratory system; ΔP = driving pressure; PEEP = positive end-expiratory pressure; T_0 _= time at baseline; T_20/30 _= time when positive end-expiratory pressure at 20 cmH_2_O and driving pressure at 30 cmH_2_O.

The RM was performed as shown in Figure 1. First, baseline measurements (time = T_0_) were taken at the settings determined by the respective attending physician in the pressure control mode. Settings were noted and Cdyn was calculated via the Open Lung Tool™. Thereafter, PEEP was set at 20 cmH_2_O and the lungs were recruited by stepwise increases of the driving pressure up to 30 cmH_2_O (time = T_20/30_).

**Figure 2:**
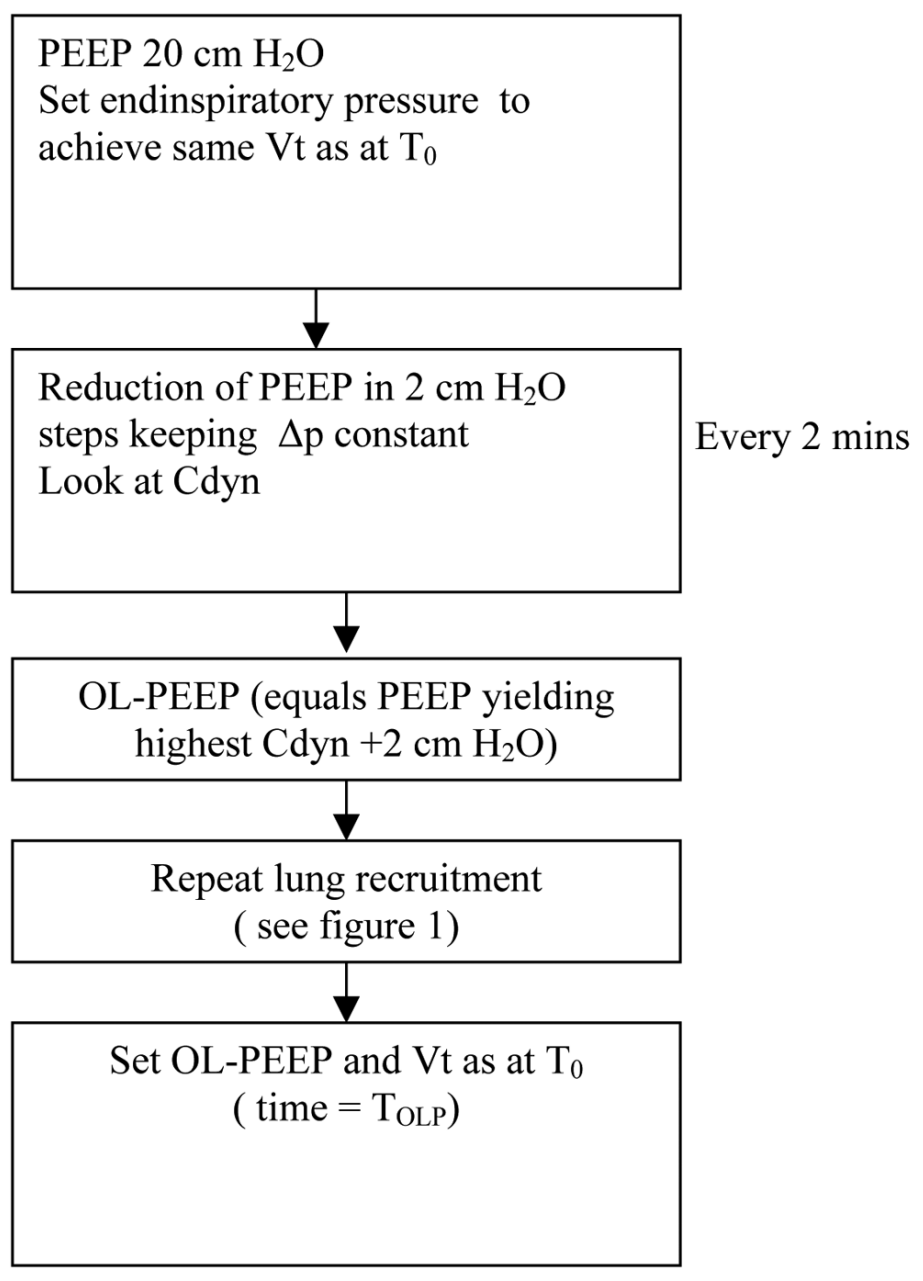
Positive end-expiratory pressure titration using the Open Lung Tool™. Cdyn = dynamic compliance of the respiratory system; OL-PEEP = open lung positive end-expiratory pressure; ΔP = driving pressure; PEEP = positive end-expiratory pressure; T_0 _= time at baseline.

Following RM, OL-PEEP was titrated as shown in Figure 2. PEEP was kept constant at 20 cmH_2_O, but EIP was reduced in order to achieve about the same Vt as at baseline. Every two minutes, PEEP was reduced in steps of 2 cmH_2_O keeping driving pressure constant and recording Cdyn. OL-PEEP was defined as the PEEP yielding highest Cdyn +2 cmH_2_O. The RM (Figure 1) was repeated and OL-PEEP was set along with the EIP that resulted in the same Vt as at T_0 _(time = T_OLP_). All measurements were carried out in the pressure-controlled mode, without changing fraction of inspired oxygen (FiO_2_) or RR.

### Protocol

Haemodynamic and transoesophageal echocardiography data were recorded at three time points: at baseline (T_0_), two minutes after the final step of the RM at a PEEP of 20 cmH_2_O and a driving pressure of 30 cmH_2_O (T_20/30_) (Figure 1) and at OL-PEEP (T_OLP_) (Figure 2). Gas-exchange and respiratory data were collected at T_0 _and T_OLP_, but not during the short-lived high pressure RM.

### Statistics

All data are presented as mean ± standard deviation. To test normal distribution, the Kologomorow-Smirnov and the Anderson-Darling tests were used. To analyse statistical differences paired sample *t*-test was applied if two times points were compared, otherwise the analysis of variance for repeated measurements was used. Bonferroni's correction to control for the number of tests was applied when indicated.

To investigate the relationship between the observed variables, Scheffe's test was performed. SAS version 9.1.3 (SAS institute, Cary, NC, USA) was used for statistical analysis. All statistical tests were only used to describe the findings.

## Results

### Demographics

After fulfilling the inclusion criteria, 12 patients were enrolled over a period of 1.5 years in a prospective autocontrol clinical trial. The demographic data of the patients are presented in Table 1.

**Table 1:** Patient characteristics

**Patient No.**	**Diagnosis**	**BMI**	**MV prior to inclusion, hours**	**PaO_2_/FiO_2_**	**PEEP**	**S/D**
1	Sepsis	24	62	170	18	S
2	Sepsis	27	58	98	16	D
3	Pneumonia	31	39	54	15	S
4	Pneumonia	23	55	61	12	D
5	Pneumonia	29	84	103	12	D
6	Pneumonia	32	69	162	17	S
7	Pneumonia	25	45	188	10	S
8	Pneumonia	31	66	83	14	S
9	Pneumonia	26	59	144	15	S
10	Pneumonia	23	76	151	14	S
11	Sepsis	27	56	102	10	S
12	Pneumonia	25	44	49	16	D

BMI = body mass index; D = died; FiO_2 _= fraction of inspired oxygen; MV = mechanical ventilation; PaO_2 _= partial pressure of arterial oxygen; PEEP = positive end-expiratory pressure; S = survived.

### Respiratory variables

At baseline conditions, patients were on a lung protective strategy with low tidal volume (5.4 ± 0.8 ml/kg IBW) and high PEEP (14 ± 3 cmH_2_O). Compared with baseline, RM followed by OL-PEEP ventilation increased oxygenation (PaO_2_/FiO_2 _at T_0 _120 ± 59 vs 146 ± 64 mmHg at T_OLP_, *P *< 0.005; Table 2). From T_0 _to T_OLP_, PEEP was increased in five patients and decreased in seven patients, leaving mean PEEP unchanged (14 ± 3 cmH_2_O).

**Table 2:** Respiratory variables presented as mean ± standard deviation

	**T_0_**	**T_OLP_**
PH	7.22 ± 0.2	7.22 ± 0.3
PaO_2_/FiO_2 _(mmHg)	120 ± 59	146 ± 64^a^
PaCO_2 _(mmHg)	58 ± 22	53 ± 18
Peak inspiratory pressure (cmH_2_O)	32 ± 3	29 ± 3^a^
PEEP (cmH_2_O)	14 ± 3	14 ± 3
Dynamic compliance (ml/cmH_2_O)	23 ± 5	27 ± 6^a^
Tidal volume (ml/kg)	5.4 ± 0.8	5.6 ± 0.7
Respiratory rate (breaths/min)	19 ± 3	19 ± 3

^a^*P *< 0.05 compared with T_0_.

FiO_2 _= fraction of inspired oxygen; PaCO_2 _= partial pressure of arterial carbon dioxide; PaO_2 _= partial pressure of arterial oxygen; PEEP = positive end-expiratory pressure; T_0 _= time at baseline; T_OLP _= time at open lung-positive end-expiratory pressure.

**Figure 3:**
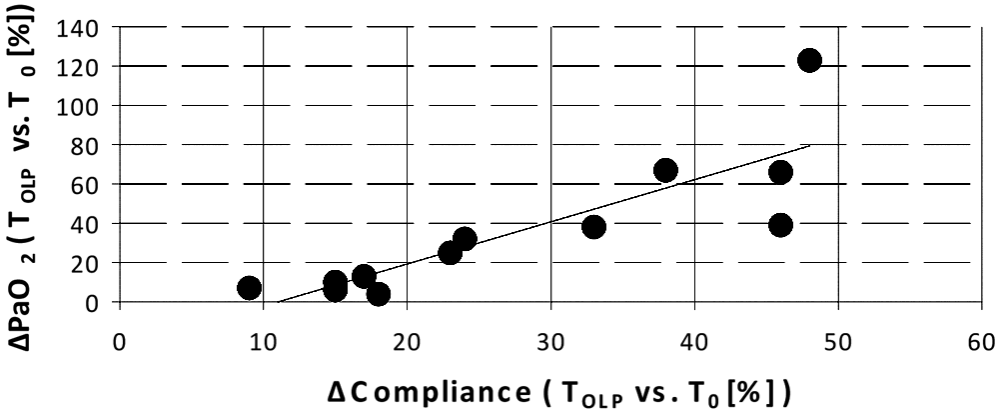
Correlation graph of percentage difference of dynamic compliance and percentage change in PaO_2 _from T_0 _to T_OLP_. *P *< 0.05, r = 0.62. PaO_2 _= partial pressure of arterial oxygen; T_0 _= time at baseline; T_20/30 _= time when positive end-expiratory pressure at 20 cmH_2_O and driving pressure at 30 cmH_2_O.

From T_0 _to T_OLP_, Cdyn significantly improved (23 ± 5 vs 27 ± 6 ml/cmH_2_O, *P *< 0.05), resulting in lower peak inspiratory pressures (29 ± 3 at T_OLP _vs 32 ± 3 cmH_2_O at T_0_, *P *< 0.05). There was a significant correlation between the percentage changes from T_0 _to T_OLP _in oxygenation and Cdyn (r = 0.62, *P *< 0.005; Figure 3). In addition, there was a significant correlation between the changes in Cdyn and the changes in partial pressure of arterial carbon dioxide (PaCO_2_) from T_0 _to T_OLP _(r = -0.52, *P *< 0.05). Tidal volume, PaCO_2 _and pHa remained constant throughout the study.

### Haemodynamics

Lung recruitment and PEEP titration using the stepwise approach guided by the Open Lung Tool™ did not result in significant haemodynamic disturbances as indicated by changes in HR, MAP or CI (Table 3). Combining CI and MAP, cardiac power index (CI*MAP*0.022 [W/m^2^]) [26] transiently decreased during lung recruitment (0.6 ± 0.2 at T_0 _vs 0.5 ± 0.2 W/m^2^at T_20/30_, *P *< 0.05), but recovered and even exceeded baseline values at T_OLP _(0.7 ± 0.2 W/m^2 ^at T_20/30_, *P *< 0.005).

**Table 3:** Haemodynamic data derived from PiCCO™-monitoring

	**T_0_**	**T_20/30_**	**T_OLP_**
Heart rate (beats/min)	86 ± 20	89 ± 20	85 ± 18
Mean arterial pressure (mmHg)	79 ± 13	71 ± 17	79 ± 13
Central venous pressure (mmHg)	22 ± 6	26 ± 4	21 ± 5
Cardiac index (l/min/m^2^)	3.3 ± 0.7	3.1 ± 0.9	3.4 ± 0.6
Cardiac power index (W/m^2^)	0.58 ± 0.17	0.48 ± 0.19	0.66 ± 0.18^b^
Stroke volume index (ml/m^2^)	37 ± 9	34 ± 14	40 ± 10
Stroke volume variance (ml)	14 ± 7	17 ± 5	13 ± 4
Intrathoracic blood volume index (ml/m^2^)	883 ± 215	-	898 ± 241
Extravascular lung water index (ml/kg/m^2^)	16 ± 9	-	17 ± 10

^a^*P *< 0.05 compared with T_0_; ^b^*P *< 0.05 compared with T_20/30_; Data are presented as mean ± standard deviation.

PiCCO™ = Pulse Contour Cardiac Output Monitor; T_0 _= time at baseline; T_20/30 _= time when positive end-expiratory pressure at 20 cmH_2_O and driving pressure at 30 cmH_2_O; T_OLP _= time at open lung-positive end-expiratory pressure.

### Transoesophageal echocadiography

**Figure 4:**
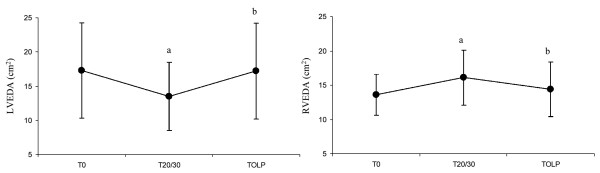
End-diastolic area changes of the left and right ventricle from T_0 _to T_20/30 _to T_OLP_. *P < 0.05 compared with T_0; _^†^*P *< 0.05 compared with T_20/30_. LVEDA = left ventricular end-diastolic area; RVEDA = right ventricular end-diastolic area; T_0 _= time at baseline; T_20/30 _= time when positive end-expiratory pressure at 20 cmH_2_O and driving pressure at 30 cmH_2_O; T_OLP _= time at open lung-positive end-expiratory pressure.

**Figure 5:**
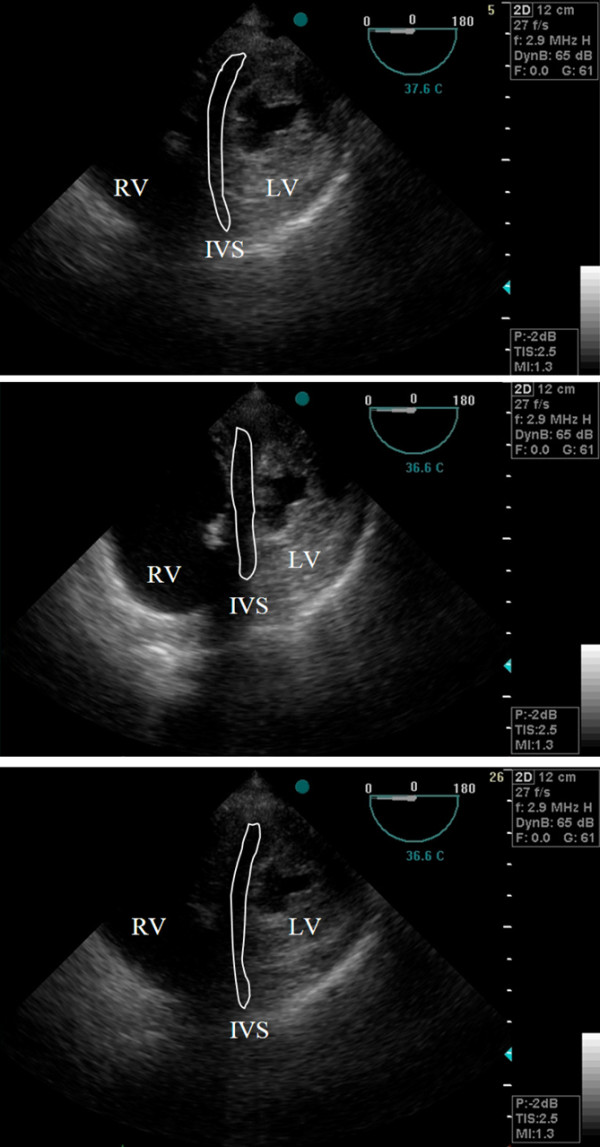
**(a)** End-systolic transgastric midpapillary views obtained at baseline, **(b)** during the recruitment manoeuvre and **(c)** during open lung positive end-expiratory pressure. Note the massive dilation of the right ventricle (RV), causing acute leftward shift of the interventricular septum (IVC) and compression of the left ventricle (LV; d-shaped) during the recruitment manoeuvre.

Maximal inferior vena cava (IVC) diameter decreased during RM (2.2 ± 0.4 at T_0 _vs 1.8 ± 0.4 cm at T_20/30_, *P *< 0.05), although minimum IVC diameter and superior vena cava diameters remained unchanged (Table 4). Right ventricular Tei index showed pathological values (> 0.4) in 6 of 12 patients at baseline. During RM, RV Tei index further deteriorated (0.39 ± 0.11 at T_0 _vs 0.42 ± 0.1 at T_20/30_, *P *< 0.05), but improved at T_OLP _(0.35 ± 0.11, *P *< 0.05). Right ventricular end-diastolic area increased during the RM (13.6 ± 3 at T_0 _vs 16.1 ± 4 cm^2 ^at T_20/30_, *P *< 0.005) and returned to baseline values at OL-PEEP. Left ventricular end-diastolic area (17.3 ± 7 at T_0 _vs 13.5 ± 5 cm^2 ^at T_20/30_, *P *< 0.05) significantly decreased during RM as did left ventricular end-diastolic septal to lateral diameters (4.2 ± 0.9 at T_0 _vs 3.6 ± 0.9 cm at T_20/30_, *P *< 0.05). At OL-PEEP, left ventricular end-diastolic area and diameters equalled baseline values. The respective changes in right ventricular and left ventricular end-diastolic areas are displayed in Figure 4. Figure 5 shows an echocardiographic example of the end-diastolic right ventricular enlargement during the RM, causing acute leftward septal shift and compression of the left ventricle.

**Table 4:** Echocardiographic data presented as mean ± standard deviation

	**T_0_**	**T_20/30_**	**T_OLP_**
Maximum diameter vena cava inferior (cm)	2.2 ± 0.44	1.8 ± 0.4	2.14 ± 0.35
Minimum diameter vena cava inferior (cm)	1.52 ± 0.37	1.3 ± 0.47	1.44 ± 0.36
Maximum diameter vena cava superior (cm)	1.92 ± 0.43	1.85 ± 0.65	1.8 ± 0.55
Minimum diameter vena cava superior (cm)	1.3 ± 0.39	1.18 ± 0.44	1.1 ± 0.35
Diameter left ventricle anterior-posterior end-systolic (cm)	3.2 ± 1.5	2.9 ± 1.3	3.1 ± 1.4
Diameter left ventricle anterior-posterior end-diastolic (cm)	4.5 ± 1.4	4.3 ± 1.2	4.8 ± 1.5
Diameter left ventricle septal-lateral end-systolic (cm)	2.8 ± 0.9	2.5 ± 0.9	2.7 ± 0.8
Diameter left ventricle septal-lateral end-diastolic (cm)	4.2 ± 0.9	3.5 ± 1^a^	4.2 ± 1.1^b^
End-systolic area of left ventricle (cm^2^)	8.3 ± 5	6.8 ± 4.1	7.2 ± 3.7
End-diastolic area of left ventricle (cm^2^)	17.3 ± 7	13.5 ± 5^a^	17.2 ± 7^b^
Left ventricular ejection fraction (%)	66 ± 14	60 ± 11^a^	69 ± 10^b^
End-systolic area of right ventricle (cm^2^)	8.1 ± 5	7.7 ± 3	7.8 ± 4
End-diastolic area of right ventricle (cm^2^)	13.6 ± 3	16.1 ± 4^a^	13.4 ± 4^b^
Right ventricular Tei index (%)	39 ± 11	42 ± 10^a^	36 ± 11^a/b^

^a^*P *< 0.05 compared with T_0_; ^b^*P *< 0.05 compared with T_20/30_.

Right ventricular Tei index was calculated as the sum of the isovolumic contraction time and the isovolumic relaxation time, divided by ejection time. T_0 _= time at baseline; T_20/30 _= time when positive end-expiratory pressure at 20 cmH_2_O and driving pressure at 30 cmH_2_O; T_OLP _= time at open lung-positive end-expiratory pressure.

## Discussion

This study shows that a standardised open lung strategy consisting of a RM followed by a decremental PEEP trial was effective in improving respiratory system mechanics and oxygenation in patients fulfilling standard ARDS criteria [21,27] while already being ventilated with low tidal volume and high PEEP. No clinically significant haemodynamic compromise occurred during the stepwise RM. During the RM, transoesophageal echocardiography revealed increased right ventricular stress and strain, indicated by an increase in right ventricular Tei index, an increase in right ventricular end-diastolic area and a consecutive acute leftward shift of the interventricular septum, resulting in a decreased septal to lateral left ventricular end-diastolic diameter and left ventricular end-diastolic area. During OL-PEEP ventilation, however, right ventricular function assessed by the Tei index was improved compared with baseline conditions with left ventricular function being unchanged.

Two different methods have been proposed as the possible approaches to recruiting the lung: high-level continuous positive airway pressure (CPAP) [28,29] and pressure control ventilation with high peak and end-expiratory pressure [30-33]. As animal models showed less cardiovascular compromise with the latter approach [34], pressure control ventilation may be considered the optimal approach to lung recruitment [35]. Accordingly, in this study we used the pressure control strategy, applying a stepwise increasing peak inspiratory pressure up to 50 cmH_2_O at a high level of PEEP, similar to the approach used by Villagra and colleagues [33].

We observed a mean percentage increase in PaO_2_/FiO_2 _of 22% following the RM and decremental PEEP trial. Furthermore, the improvement in oxygenation was associated with an increase in the dynamic respiratory compliance, suggesting the presence of alveolar recruitment.

The oxygenation response in our study was in line with that reported by Villagra and colleagues [33] but modest compared with the study by Grasso and colleagues [28]. This can be explained by different types of patients, the ALI/ARDS onset time and ventilatory setting. In particular, it should be considered that our patients were on a lung protective strategy with low tidal volume and high PEEP (mean PEEP at baseline of 14 cmH_2_O), which is likely to result in a lesser improvement in respiratory function after RMs.

The primary complications possibly occurring during RMs are barotrauma and haemodynamic compromise [16,17,36,37]. RMs may impair haemodynamics, most commonly assessed by MAP or cardiac output, by two main mechanisms [8]. First, as the lung is recruited, high airway pressure can more readily be transmitted across the lung parenchyma to the pleural space, impeding venous return and thus decreasing right ventricular preload. Second, high alveolar pressure may increase pulmonary vascular resistance and right ventricular afterload. A recent systematic review [37] revealed hypotension (12%) and desaturation (9%) as the most frequent complications, although serious adverse events such as barotrauma were rare (1%). Given these side effects and the lack of information on the influence on clinical outcome, the authors neither recommend nor discourage RMs at this time. The latter point is especially important, as the effect of RMs is relatively short-lived and RMs must be repeated several times a day in order to maintain open lung ventilation.

The study presented here, albeit small, did not reveal major complications. In particular, we did not observe any significant decrease in MAP, stroke volume or CI during the RMs. Cardiac pumping capability, however, assessed by the cardiac power index, which combines both pressure and flow domains of the cardiovascular system, decreased. These findings of relative haemodynamic stability during the RMs are in line with those reported in the ARDS Network study [4,38] showing a 10.6 ± 1.2 mmHg decrease in systolic blood pressure during lung recruitment manoeuvre using CPAP over 5 to 10 seconds at 35 to 40 cmH_2_O and the study by Borges and colleagues [30] using peak airway pressures up to 60 cmH_2_O, where none of the patients investigated experienced haemodynamic compromise during the RMs.

Despite maintained blood pressure and CI, the RMs induced an acute cardiac stress test as evidenced by transoesophageal echocardiography. This implies that monitoring haemodynamics using arterial pressure and cardiac output in clinical practice is likely to miss specific changes in venous return and/or right ventricular loading conditions. Echocardiographic assessment of vena cava diameters, which remained unchanged during the RMs except for maximum IVC diameter, revealed maintained venous return in the present study. The patients in our study were at the lower limits of normovolaemia, as indicated by a mean intrathoracic blood volume index of 883 ml/m^2 ^and a stroke volume variation of 14%, suggesting that RMs by pressure control ventilation can safely be performed at low normal volume status without the need to induce potentially detrimental hypervolaemia. The importance of the intravascular volume status during the recruitment manoeuvre has been specifically addressed by Nielsen and colleagues [15] in a porcine lung-lavage model: using transoesophageal echocardiography, they showed left ventricular compromise resulting in a drop in cardiac output during lung recruitment by sustained inflation (40 cmH_2_O of CPAP for 30 seconds), which was accentuated by hypovolaemia and attenuated by hypervolaemia. Taken together, these findings underscore the need to ensure an adequate intravascular volume status before attempting RMs.

Although venous return was maintained, the RMs, by inducing lung inflation, most probably increased pulmonary vascular resistance [39], thus increasing right ventricular afterload. This increase in right ventricular afterload could be assessed echocardiographically by the increase in right ventricular Tei index and the increase in right ventricular end-diastolic diameter with a consecutive, acute leftward septal shift, reducing left ventricular size. These findings were not as severe as those seen in the study by Nielsen and colleagues [16], when 40 cmH_2_O of CPAP for 10 to 20 seconds was applied to patients following cardiac surgery. Recorded in patients with healthy lungs, these manoeuvres most probably resulted in severe lung overinflation, making the acute right ventricular overload very predictable [17,39]. The situation may be different in patients with ALI/ARDS, when high airway pressure is less readily transmitted across the lung parenchyma to the pleural space, causing less impairment of venous return and cardiac output [8]. This, in addition to the fact that pressure control ventilation instead of sustained inflation was used, may explain the lesser degree of right ventricular dysfunction caused by the RM in the present study.

Although the RM, which is needed as part of the open lung procedure, presents a cardiac stress test mainly due to an acute increase in right ventricular afterload, at OL-PEEP right ventricular function as assessed by the Tei index was even improved compared with baseline settings. Left ventricular function at OL-PEEP was comparable with baseline.

In order to explain these findings, we hypothesise that better oxygenation at lower peak pressure (i.e. better compliance) after a RM and decremental PEEP trial has shifted the ventilation to the deflation limb of the pressure/volume envelope, causing ventilation to take place at higher lung volumes. If this results in higher end-expiratory lung volumes approaching normal FRC, but not causing overdistention, pulmonary vascular resistance will fall due to the U-shaped relation between pulmonary vascular resistance and lung volume. A recent computed tomography study in lung-injured pigs showed that PEEP at which compliance was maximal resulted in the best compromise between recruitment and overinflation [40], which might help to explain the improvement in right ventricular function observed in the present study. These findings are also in keeping with the results from Reis Miranda and colleagues [41], who showed that ventilation according to the open lung concept consisting of high PEEP following a RM did not increase right ventricular outflow impedance compared with conventional ventilation with lower PEEP. The authors propose that resolution of atelectasis due to the RM decreases right ventricular outflow impedance and thus counterbalances the potentially detrimental effects of high PEEP on right ventricular function [8]. In fact, Duggan and colleagues showed that atelectasis causes significant increases in right ventricular afterload and that this may even lead to right ventricular failure in healthy rats [42].

To better interpret our results, some limitations need to be addressed. A relatively small number of patients were included in the study due to a selection of more severe patients with early ARDS and absence of haemodynamic instability and without significant arrythmias. As we investigated a specific RM, it is possible that different results could be obtained by using other manoeuvres. Finally, the measurements were made only at the end of the recruitment procedure, which overall lasts for six minutes. The clinical consequence of the RM may not be trivial and in order to keep the lung open the RM must be repeated several times a day in clinical practice.

## Conclusions

In conclusion our study demonstrates that standard recruitment manoeuvres during protective ventilation can be associated with haemodynamic changes not revealed by conventional haemodynamic monitoring. A decremental titration of PEEP aimed to yield maximum dynamic compliance was associated with an improvement in oxygenation, dynamic respiratory system compliance and unloading the right ventricle while not affecting the left ventricle.

## Key messages

• A standardised open lung strategy consisting of a recruitment manoeuvre followed by a decremental OL-PEEP trial inproves oxygenation and respiratory system compliance in patients with ARDS already ventilated in a lung protective mode.

• Although major haemodynamic indices remain unchanged, transoesophageal echocardiography reveals increased right ventricular stress and strain during the recruitment phase.

• Compared with baseline values, right ventricular function is improved at OL-PEEP.

## Abbreviations

ALI: acute lung injury; ARDS: adult respiratory distress syndrome; Cdyn: dynamic compliance of the respiratory system; CI: cardiac index; CPAP: continuous positive airway pressure; EIP: end-inspiratory pressure; FiO_2_: fraction of inspired oxygen; FRC: functional residual capacity; IBW: ideal body weight; IVC: inferior vena cava; MAP: mean arterial pressure; OL-PEEP: open lung positive end-expiratory pressure; PaCO_2_: partial pressure of arterial carbon dioxide; PaO_2_: partial pressure of arterial oxygen; PEEP: positive end-expiratory pressure; PiCCO: Pulse Contour Cardiac Output Monitor; RM: recruitment manoeuvre; RR: respiratory rate; T_0_: time at baseline; T_20/30_: time when positive end-expiratory pressure at 20 cmH_2_O and driving pressure at 30 cmH_2_O; T_OLP_: time at open lung positive end-expiratory pressure; VILI: ventilator-induced lung injury; Vtinsp: inspiratory tidal volume.

## Competing interests

The authors declare that they have no competing interests.

## Authors' contributions

CG, GW, PP and TL participated in the study design. CG, GW and TL performed the study. CG and TL processed the data and performed the statistical analysis. TL and PP wrote the manuscript. All authors read and approved the final manuscript.
